# Evidence for the Neuronal Expression and Secretion of Adiponectin

**DOI:** 10.3390/cells11172725

**Published:** 2022-09-01

**Authors:** Azénor Abgrall, Ghislaine Poizat, Marianne Prevost, Laure Riffault, Laura De La Barrera, Rita Hanine, Katarina Djordjevic, Yacir Benomar, Mohammed Taouis

**Affiliations:** Paris-Saclay Institute of Neurosciences (NeuroPSI), University of Paris-Saclay, CNRS, F-91400 Saclay, France

**Keywords:** adiponectin, hypothalamus, signaling, neurons

## Abstract

Peripheral adiponectin acts on the hypothalamus to inhibit energy expenditure and increase food intake through its receptors AdipoR1 and adipoR2. The hypothalamic expression of adiponectin is poorly documented. We hypothesize that whether hypothalamic adiponectin is confirmed, its expression and secretion could be regulated as peripheral adiponectin. Thus, in the present work, we aim to determine whether adiponectin is expressed in the hypothalamus and in two neuronal cell lines and investigate the potential mechanisms regulating its neuronal expression. Using immunohistochemistry, we show that adiponectin is expressed in the mediobasal hypothalamic neurons of mice. Adiponectin expression is also evidenced in two neuronal cell lines mHypo POMC (an adult mouse hypothalamic cell line) and SH-SY5Y (human neuroblastoma). The neuronal expression of adiponectin is increased in response to rosiglitazone treatment (a PPARγ agonist) and FGF21 and is decreased in insulin-resistant neurons. Furthermore, we show that adiponectin expressed by mHypo POMC neurons is secreted in a culture medium. Adiponectin also diminished the resistin-induced IL6 expression in SIMA9 cells, a microglia cell line. In conclusion, we evidenced the hypothalamic expression of adiponectin and its regulation at the neuronal level.

## 1. Introduction

The hypothalamus, particularly the mediobasal hypothalamus (MBH), plays a crucial role in detecting and integrating metabolic and endocrine signals conveyed by the blood. This is facilitated by its atypical cellular organization and the presence of fenestrated capillaries in the median eminence (EM) and local tanycytes. The latest transport peripheral peptides from the ME to the third ventricle then reaching the MBH cells [1,2], where an adequate hypothalamic response to the body’s energy needs will be generated. Impaired communication between the MBH and the periphery is associated with a wide range of metabolic disorders, including obesity and type 2 diabetes. One of the mechanisms contributing to these diseases is the development of inflammation of the MBH in response to the consumption of a high-fat diet (HFD) and subsequent changes in plasma hormone levels [1]. Indeed, long-term consumption of an HFD alters the expression of hormones in adipose tissue called adipokines and there is in particular an increase in the expression of resistin and leptin associated with the decrease in adiponectin expression [3,4,5,6]. HFD-induced hyperleptinemia induces hypothalamic leptin resistance, thus altering energy homeostasis [7]. Increased resistin secretion, produced by adipose tissue in rodents and by immune cells in humans, induces insulin resistance associated with systemic and tissue inflammation [8]. We have previously shown that resistin at the central level acts via the TLR4 receptor and causes reactive gliosis associated with the increased expression of pro-inflammatory cytokines at the hypothalamic level [9,10]. We have also shown that resistin at the hypothalamic level inhibits insulin signaling as well as that of adiponectin and FGF21, inducing hypothalamic and peripheral insulin resistance [9,10]. This reveals crosstalk at the hypothalamic level involving resistin, insulin, adiponectin, and FGF21 signaling pathways, which would be involved in HFD-induced neuroinflammation and insulin resistance. Besides, it has been shown that resistin is expressed by the MBH and its role is still unknown, and this raises the question concerning adiponectin and whether MBH expresses local adiponectin, which may be involved in potential crosstalk with resistin and FGF21 within the MBH to regulate hypothalamic insulin responsiveness and the expression of pro-inflammatory cytokines. Indeed, at the peripheral level, adiponectin generated by the white adipose tissue acts as an antidiabetic adipokine [11] through the promotion of insulin action, implicating different mechanisms, as for instance the suppression of hepatic neoglucogenesis, the augmentation of fatty acid β-oxydation [12,13,14], and the augmentation of hepatic IRS-2 expression [15]. Adiponectin acts through two receptors, adiponectin receptors 1 and 2, activating the AMPk and PPARα pathways [16,17]. Besides, it has been shown that in obese [18] and diabetic subjects the circulating levels of adiponectin are low [19,20]. Furthermore, the expression of adiponectin by adipocytes is activated by a hepatokine, FGF-21 (fibroblast growth factor 21), via its action on the FGFR/βKlotho receptor/co-receptor complex in adipocytes, leading to the activation of the PPARγ pathway. PPARγ binds to a nuclear PPRE responsive element located on the adiponectin promoter, leading to adiponectin expression [21]. The expression of adiponectin in adipose tissue can also be induced by thiazolidinediones, a family of molecules used in treatments against insulin resistance and type II diabetes [22] such as rosiglitazone, a PPARγ agonist known for its insulin-sensitizing role. Indeed, it has been demonstrated in studies carried out in humans that treatment with rosiglitazone increases the level of expression of adiponectin in healthy subjects, but also in diabetic or obese subjects with an improvement in their insulin sensitivity [23].

In the hypothalamus, adiponectin acts through its receptors (AdipoR1 and R2) to activate AMPK phosphorylation, leading to the augmentation of food intake and the diminution of energy expenditure [24]. However, adiponectin expression in the brain, and more specifically in the hypothalamus, is poorly documented. Indeed, a limited number of studies reported the expression of adiponectin in the hypothalamus of mice, beavers, or female pigs [25,26,27]. Even though a study reported the presence of adiponectin in the brain, it accumulated in the vascular endothelial cells and they concluded that adiponectin is not expressed by hypothalamic neural cells [28]. In the present work, we show that adiponectin is expressed in the hypothalamus of mice and in mHypo neuronal cell line derived from the adult mouse hypothalamus. Furthermore, we find that the expression of neuronal adiponectin is regulated as evidenced by its upregulation in response to rosiglitazone or FGF21 and downregulated in response to insulin resistance.

## 2. Materials and Methods

### 2.1. Animals

Six-week-old male C57bl/6J (Envigo, Gannat, France) mice were housed in standard conditions (12-h light/dark cycle; 22 ± 1 °C) and fed ad libitum the standard diet (SAFE, Augy, France) with free access to water. At the age of 8 weeks, the mice were fasted overnight and then sacrificed the next day either by intracardiac infusion or by decapitation. For the mice having received an intracardiac injection of PFA4%, their brains were removed and post-fixed in a 4% PFA solution overnight at 4 °C. They were then placed the next day in a 20% sucrose solution and then, 24 h later, frozen in isopentane at −40 °C. The brains were then stored at −80 °C before being cut up in order to perform immunohistochemistry. For mice that had been decapitated, the hypothalami were recovered, immediately frozen, then stored at −80 °C until RNA extraction. All experimental procedures were performed according to the institutional guidelines for animal use specified by the European Union 621 Council Directive (2010/63/EU) and approved by the French Ethics Committees for the Care and Use of Experimental Animals (C2EA, 59 Comité Paris Centre et Sud; authorization n° 27899).

### 2.2. Cell Culture

To investigate adiponectin neuronal expression and regulation we have used two cell lines: mHYPO POMC GFP (Cedarlane, Burlington, NC, USA), a neuronal cell line derived from adult mouse hypothalamic, and SH-SY5Y (DSMZ, Village-Neuf, France), a cell line derived from human neuroblastoma. mHYPO-POMC-GFP cells were cultured in Dulbecco’s modified Eagle’s medium (DMEM) 4.5 g/L glucose supplemented with 10% fetal calf serum (FCS), 1% glutamine, and 1% penistreptomycin. SH-SY5Y cells were cultured in RPMI medium (Gibco) supplemented with 10% FCS, 1% glutamine, and 1% penistreptomycin. To study the regulation of adiponectin expression in a situation of insulin resistance or in the presence of FGF21, mHYPO POMC cells were treated with insulin (100 nM) [9] or FGF21 (10 nM) [29] for 16 h in serum-free media. In order to study the impact of rosiglitazone, mHYPO POMC cells were incubated in the presence of rosiglitazone at 0.5 µM, 1 µM, or 10 µM [23] for 24 h or 48 h in serum-free media (SHSY5Y) or media complemented with 1% serum (mHYPO POMC). For the study concerning IL6 expression, serum-starved cells were incubated in the presence or absence of both resistin (70 ng/mL) and adiponectin (1 μg/mL) for 16 h. All treatments were stopped by aspiration of the media and phosphate saline buffer (PBS) washes. For Western blot analyses, cells were lysed in a 3X lysis buffer containing SDS (sodium dodecyl sulfate), DTT (dithiothreitol), glycerol, and bromophenol blue. For RT-qPCR analyses, cells were suspended in TRIzol for RNA extraction.

### 2.3. RNA Extraction and Quantitative RT-PCR

Total RNAs from mice hypothalami or neuronal cells were isolated using TRIzol reagent and quality checked with NanoDrop spectrophotometer (Ozyme). One microgram of RNA was reverse transcribed following the manufacturer’s recommendations (qScript cDNA Synthesis Kit-95047-QuantaBio) and the cDNAs were submitted to quantitative real-time PCR (polymerase chain reaction) using SYBR Green QPCR (Power Up SYBR Green Master Mix Kit-A25778-Appliedbiosystems) or classic PCR (Phusion high fidelity master mix-M0531S-NEB). For each amplification, specific primers were used (sequences in Supplementary Figure S1). The qPCR is performed according to the manufacturer’s recommendations. Adiponectin or IL6 transcripts were quantified relative to those of GAPDH transcripts, and relative to the control (CT), by using the ΔΔCt method and experimentally ascertained amplification efficiencies for each reaction. GAPDH housekeeping gene has been previously tested and used in these cellular models. All samples were measured in duplicates and negative controls were performed in reverse transcription and amplification steps to ensure the specificity of the measured signal. Concerning classic PCR, adiponectin transcript expression was compared to 18S expression also used as a good housekeeping gene in tested models. A list of primers used is presented in Figure S1.

### 2.4. Western Blot

Following treatments (insulin, FGF21 or Rosiglitazone), cells were lyzed in 3X lysis buffer (1M ph6.5 Tris-HCl, 15% SDS, 60% glycerol, bromophenol blue, β-mercaptoethanol) and heated for 5 min at 95 °C. The proteins were then subjected to SDS-PAGE in a 12% acrylamide gel and then electrotransferred to polyvinylidene fluoride (PVDF) membranes (Immobilon). Following blocking, membranes were incubated in the presence of anti-rabbit adiponectin primary antibody diluted in the blocking solution (1:1000, GTX112777) [30]. The membranes were then washed with 0.1% TBS/Tween (10X TBS buffer, milliQ water, Tween) and incubated for 1 h with a solution of anti-rabbit HRP secondary antibody diluted in 0.1% TBS/Tween buffer (1:15,000, Sigma, St. Louis, MA, USA). After washes, signal was revealed with WesternBright ECL HRP substrate (Advansta, Blagnac, France) for 2 min before with the Bio Rad ChemiDoc Imaging System device. Western blot quantification was performed using ImageJ software measuring adiponectin signal and normalized with tubulin. 

### 2.5. Immunohistochemistry

Mice under anesthesia were transcardially infused with 4% paraformaldehyde (PFA) solution. Brains were collected, post-fixed in PFA 4%, cryoprotected in 20% sucrose solution for 24 h, and frozen in −40 °C cooled isopentane solution. Brain coronal sections (10 µM thickness) through the hypothalamus were then subjected to standard immunohistochemistry protocol. Briefly, sections were first incubated with 50 mM NH4Cl for 20 min and blocked/permeabilized with PBS solution containing 0.1% Triton X-100, 0.2% fish gelatin, and 2% normal donkey serum for 1 h at room temperature. The brain sections were incubated overnight at 4 °C with: an anti-rabbit adiponectin primary antibody (GTX112777, recognizes both mouse and human adiponectin, Genetex, Irvine, CA, USA), diluted at 1:1000 in the blocking solution, an anti-adipoR1 primary antibody (H-001-44, recognizes both mouse and huma AdipoR1, Phoenix, AZ, USA) diluted at 1:200 or anti-goat adipoR2 (sc46751, recognizes both mouse and human AdipoR2, Santa Cruz, CA, USA) diluted 1:200. Secondary antibodies used are rabbit DyLight 550 for adiponectin and AdipoR1, and goat Alexa 488 for AdipoR2. Nuclei were counterstained with DAPI (1:3000 Sigma-Aldrich). The images are acquired from the mice hypothalamus (*n* = 5) at the level of the mediobasal hypothalamus, cortex, or hippocampus with a Zeiss Axio Imager 1 microscope combined with the technology of ApoTome 2 contrast enhancement with the 25× objective, water immersion. The images are acquired using the Zen software. All the images presented in the figures represent maximum intensity projections obtained using the ImageJ software. The specificity of adiponectin has been tested and presented in Figure S2. 

### 2.6. Immunocytochemistry 

The cells cultured on coverslips are fixed for 20 min at 37 °C in 4% PFA (paraformaldehyde), and permeabilized in PBS1X 0.1% Triton. Saturation of non-specific binding sites is achieved by adding a blocking solution (0.1% Triton 5% horse serum (NHS)-PBS) for 1 h at room temperature. The anti-adiponectin primary antibody is diluted at 1:1000 in the blocking solution and incubated overnight at 4 °C. Secondary antibody rabbit DyLight 550 has been used to reveal adiponectin staining and nuclei were counterstained with DAPI (1:3000 Sigma-Aldrich). Coverslips are then mounted on slides with a dedicated mounting medium (fluorescent mounting medium, Dako) and sealed with varnish. Images were taken with a microscope ZeissImager 1 ApoTome.2 as described for the hypothalamus immunohistochemistry analysis. The objective used is 63X, water immersion. The image shown represents a maximum intensity projection obtained using the ImageJ software.

### 2.7. Data Analysis and Statistics

All qPCR mRNA or Western blot quantification results are represented as boxplots when triplicates were present, histogram if not.

The Mann-Whitney non-parametric test has been used for all statistical analyses (SIEGEL (S.) and CASTELLAN (N.J.), 1988. Nonparametric statistics for the behavioral sciences. McGraw-Hill, New York, 399 p.) using Excel or R software. When more than 2 groups are present, two K sample permutation test was performed followed by a two-by-two comparison using nparcomp library in R software. The differences are considered significant from *p*-value <0.05.

## 3. Results

### 3.1. Expression of Adiponectin in Mouse Hypothalamus and Neuronal Cell Lines

**Figure 1:**
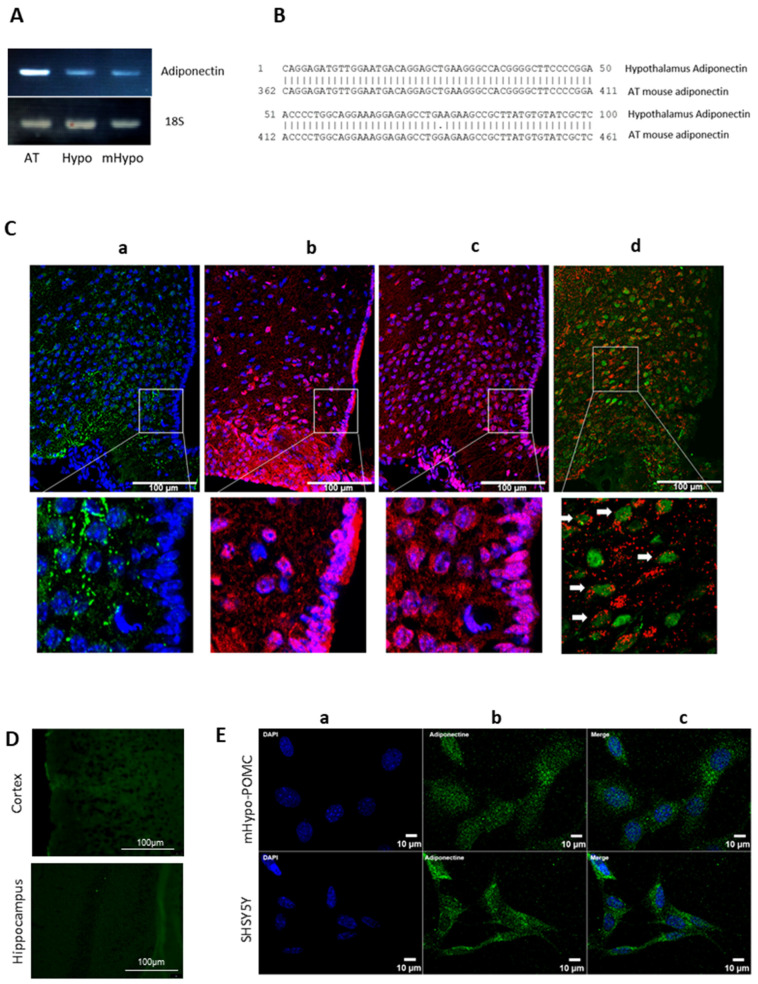
Adiponectin is expressed in hypothalamus and neuronal cells. (**A**) Total RNA from mouse adipose tissue or hypothalamus, and mHypo POMC cells were subjected to RT + PCR targeting mouse adiponectin cDNA and 18S. The amplified fragments were separated in 2% agarose gel stained with ethidium bromide. (**B**) Following purification, amplified fragments were sequenced, and hypothalamic adipose tissue adiponectin sequences were aligned. (**C**) Adiponectin expression in the hypothalamus: (a) Immunohistochemistry of adiponectin (green) protein merged with DAPI (blue), (b) of AdipoR1 protein (red) merged with DAPI (blue), (c) of AdipoR2 protein (red) merged with DAPI (blue), and (d) of adiponectin protein (red) merged with NeuN (green) and higher magnifications for each image (bottom panels). All images have been done in medio-basal hypothalamus, more precisely in arcuate nucleus of C57Bl6 mice. Full arrows indicate neuronal cells expressing adiponectin. (**D**) Immunohistochemistry of adiponectin in mice cortex and hippocampus. (**E**) Immunocytochemistry of (a) DAPI (blue), (b) adiponectin (green), and (c) merge in mouse mHypo-POMC cell line (upper panels) or SH-SY5Y cell line (bottom panels). All images have been obtained with 25× objective of Epifluorescence-Apotome microscope. Scale bars = 100 µm for hypothalamus, cortex, and hippocampus images, 10 µm for cells.

We first identified and sequenced the mRNA coding for the hypothalamic mouse adiponectin by comparing it to that of adipose tissue (reference tissue expressing adiponectin). Thus, after extracting total RNAs from visceral white adipose tissue, mouse hypothalamus, and mHYPO POMC neuronal cells, we performed RT-PCR using specific primers for mouse adiponectin. Following agarose gel separation, the three amplified fragments exhibited a similar size and are detected in the hypothalamus and mHYPO POMC neurons (Figure 1A). Fragments amplified in the hypothalamus and adipose tissue were then purified and sequenced. The sequencing confirms that the amplified fragment in the hypothalamus corresponds to adiponectin with a sequence identity of almost 100% compared to adipose tissue (Figure 1B). We then confirmed this expression by immunohistochemistry in the mouse hypothalamus (Figure 1(Ca)). We note the protein expression of adiponectin in the MBH, particularly in the arcuate nucleus, and an almost total absence in the tanycytes (Figure 1(Ca)). We also show that both AdipoR1 and AdipoR2 are expressed in the MBH and in tanycytes (Figure 1(Cb,Cc), respectively). Furthermore, the co-localization of adiponectin and NeuN staining evidence the neuronal expression of adiponectin (Figure 1(Cd)). We also demonstrated that the expression of adiponectin in the brain is mainly localized in the hypothalamus; indeed, we did not detect adiponectin expression in the cortex nor in the hippocampus (Figure 1D). 

ICC on two neuronal lines, the mHYPO POMC cells, mouse hypothalamic neuronal cell line, and the SH-SY5Y cell line derived from human neuroblastoma also showed the neuronal expression of adiponectin (Figure 1E).

### 3.2. Rosiglitazone Increases Adiponectin Expression in mHYPO POMC Neuronal Cell Line

**Figure 2:**
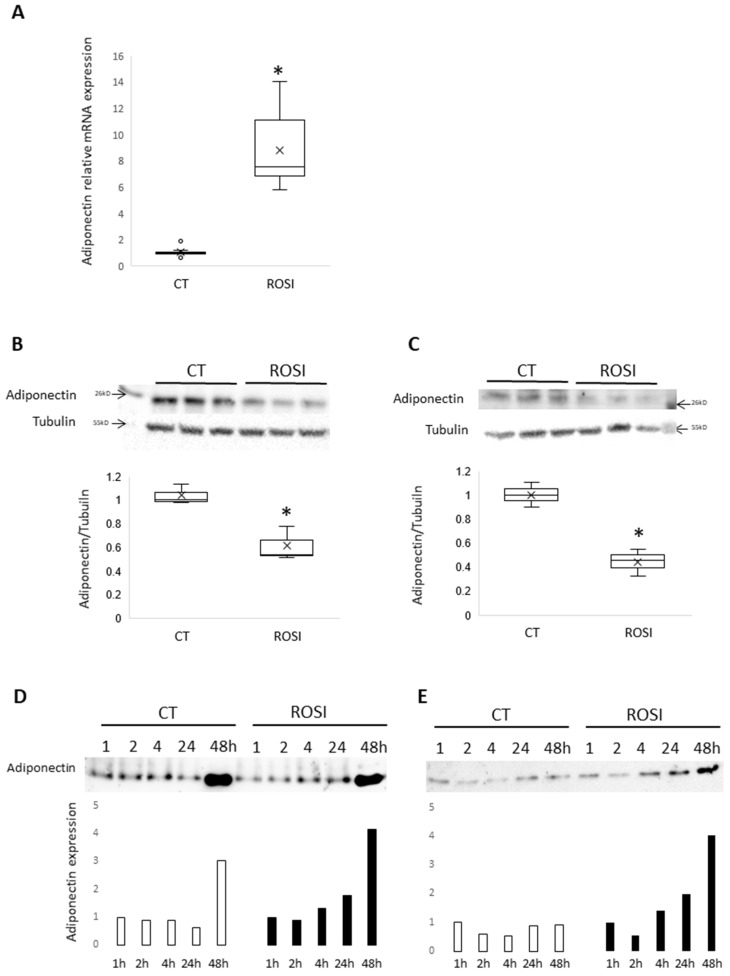
Adiponectin is regulated by rosiglitazone. (**A**) Adiponectin mRNA expression normalized to GAPDH obtained by RT-qPCR on mHypo-POMC cells stimulated 24 h with rosiglitazone 0.5 µM, *n* = 3 experiments in triplicates. (**B**) Western blot adiponectin and tubulin on mHypo-POMC cells stimulated 48 h with rosiglitazone 0.5 µM and quantification of signal intensity ratio adiponectin/tubulin, *n* = 1 experiment with triplicates. (**C**) Western blot adiponectin and tubulin on SH-SY5Y cells stimulated 48 h with rosiglitazone 1 µM and quantification of signal intensity ratio adiponectin/tubulin, *n* = 1 experiment with triplicates. (**D**) Western blot adiponectin on media of mHypo-POMC cells stimulated with or without rosiglitazone 0.5 µM during 1, 2, 4, 24, or 48 h, *n* = 1 experiment. (**E**) Western blot adiponectin on media of SH-SY5Y cells stimulated with or without rosiglitazone 1 µM during 1, 2, 4, 24, or 48 h, *n* = 1 experiment. Results are represented as boxplots showing quartiles (box), median (line), mean (x) and outliers (°) or histograms. * *p* < 0.05, statistical analyses using non-parametric Mann-Whitney test.

Since mHypo POMC neuronal cells express adiponectin, we investigated the impact of rosiglitazone, a PPARγ agonist known to increase adiponectin expression in adipocytes, on adiponectin expression in mHypo POMC cells. We show that rosiglitazone treatment with increased concentrations during 48 h significantly increases adiponectin mRNA expression and reaches the higher expression at 0.5 μM (Figure 2A). Then, the experiment has been confirmed in three independent experiences using rosiglitazone at 0.5 μM after 24 h stimulation. Furthermore, we have also tested the effect at 24 h compared to 48 h incubation in the presence of rosiglitazone and found similar data (Figure S3).

We then measured the adiponectin content of mHypo POMC and SH-SY5Y in response to rosiglitazone (0.5 μM) treatment for 48 h. In contrast with mRNA expression, rosiglitazone significantly reduces adiponectin content in both cells (Figure 2C for mHypo POMC, and Figure 2D for SH-SY5Y). To determine whether this downregulation is attributed to the secretion of adiponectin by mHypo POMC or SH-SY5Y neuronal cells, we measured adiponectin in the cell culture medium. We found that rosiglitazone (0.5 μM in mHypo-POMC, 1 µM in SH-SH5Y) increases the adiponectin secretion in a time-dependent manner with a higher secretion following 48 h of treatment (Figure 2E for mHypo POMC; Figure 2F for SH-SY5Y).

### 3.3. Insulin Resistance Inhibits Adiponectin Expression in mHYPO POMC Cells

**Figure 3:**
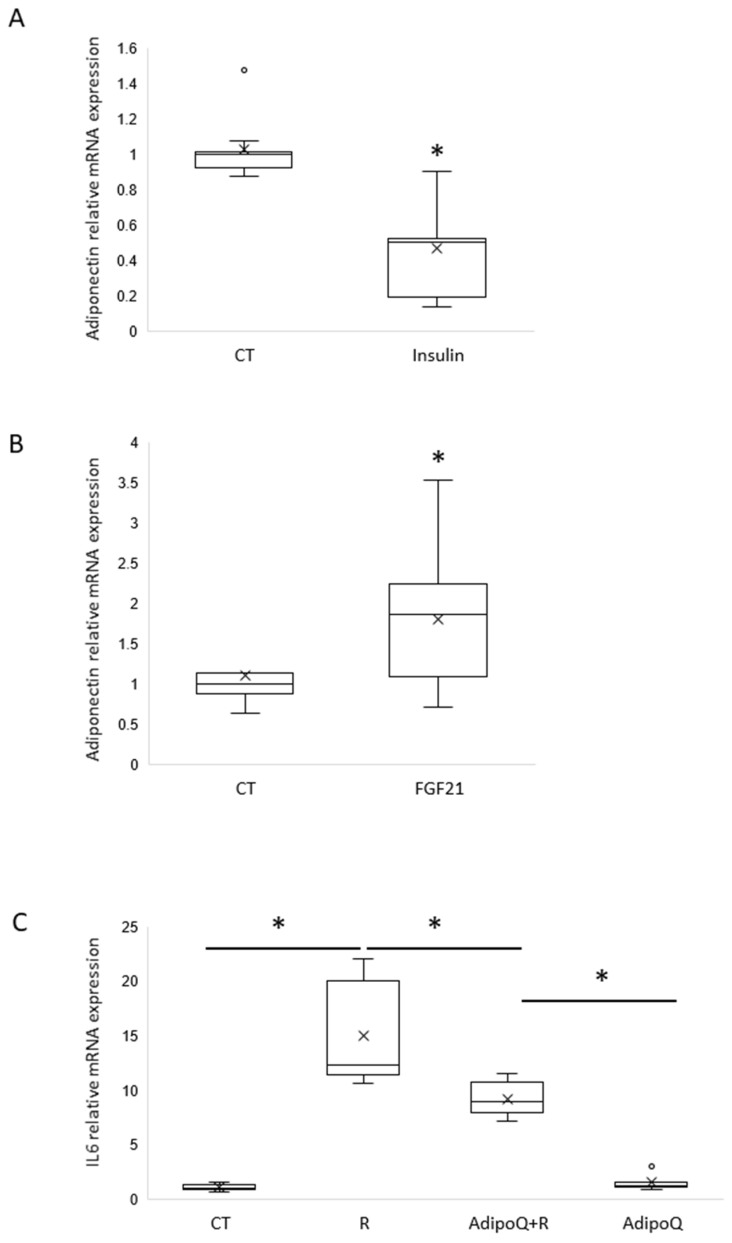
Adiponectin is regulated by insulin and FGF21 in neuronal mHypo-POMC cells and is able to regulate resistin-dependant inflammation in microglial SIMA9 cells. (**A**) Adiponectin mRNA expression normalized on GAPDH obtained by RT-qPCR on mHypo-POMC cells stimulated 16 h with insulin 100 nM, *n* = 3 experiments in triplicates. (**B**) Adiponectin mRNA expression normalized on GAPDH obtained by RT-qPCR on mHypo-POMC cells stimulated 16 h with FGF21 10 nM, *n* = 4 experiments in triplicates. (**C**) IL6 mRNA expression normalized on GAPDH obtained by RT-qPCR on SIMA9 microglial cells stimulated with or without resistin 70 ng/mL and with or without adiponectin 1 µg/mL, *n* = 3 experiments in triplicates. Results are represented as boxplots showing quartiles (box), median (line), mean (x) and outliers (°), * *p* < 0.05, statistical analyses with non-parametric Mann-Whitney test (2 groups) or two K sample permutation test followed by a two-by-two comparison using nparcomp library in R software (more than 2 groups).

To determine whether neuronal adiponectin expression is impaired in the insulin-resistant state as in adipocytes, mHypo POMC cells were overexposed to insulin for 16 h to mimic insulin resistance. This treatment led to a significant down-regulation of adiponectin in mHypo POMC cells (Figure 3A).

### 3.4. FGF21 Increases Adiponectin Expression in mHYPO POMC Cells

At the peripheral level, hepatokine FGF-21 has been shown to increase adiponectin expression by the adipocytes via PPARγ activation. We questioned whether such regulation is present in mHypo POMC neurons. Thus, mHypo POMC cells were treated with FGF21 for 16 h, and then adiponectin expression was measured by RT-qPCR. We found that FGF21 treatment significantly increases adiponectin expression (Figure 3B). 

### 3.5. Adiponectin Counteracts Resistin-Induced IL6 Expression

Based on our findings that strongly suggest that adiponectin is expressed by neurons, and based on our previous data showing that the proinflammatory resistin strongly alters adiponectin action [10], we attempted to investigate the potential anti-inflammatory action of adiponectin and its ability to counteract resistin action. For this means, SIMA9, a microglia cell line, was treated with resistin in the absence or presence of adiponectin, and the pro-inflammatory cytokine IL6 was measured by RT-PCR. We show that resistin strongly increases IL6 expression and the addition of adiponectin significantly attenuated resistin-induced IL6 expression (Figure 3C).

## 4. Discussion

The expression of adiponectin by the adipose tissue and specifically by adipocytes has been largely reported in many publications [11,17,31,32,33]. In the present work, we report the expression of adiponectin at both the mRNA and the protein levels in the hypothalamus of mice and more specifically in the MBH, and in two cell neuronal models. The expression of hypothalamic adiponectin was poorly documented [25,26,27]. We also confirmed the expression of its receptors in the MBH as we have previously reported [34]. Interestingly we also found that adiponectin as a protein is expressed in the MBH and not in cells bordering the third ventricle, mainly tanycytes. Adiponectin is also expressed in two neuronal cell lines: mHYPO POMC (adult mouse hypothalamic neuronal cell line) and SH-SY5Y (human neuroblastoma cell line). Importantly, the neuronal expression of adiponectin is regulated by the hormonal environment. Indeed, a situation of insulin resistance mimicked by insulin overexposure significantly reduces the expression of adiponectin in mHypo POMC cells and this recalls the regulation of the expression of adiponectin by adipose tissue where insulin resistance is associated with decreased adiponectin expression [19]. We have also shown that treatment with rosiglitazone for 48 h, a molecule known for its insulin-sensitizing effects through the activation of the PPARγ pathway, increases the expression of adiponectin mRNA in the mHypo POMC neuronal cell line by almost eight-fold. Moreover, the dose of rosiglitazone we used is much lower (0.5 µM) compared to that used to stimulate adipocytes. Indeed, in the 3T3-L1-mouse cell line differentiated into adipocytes, rosiglitazone at 10 μM increases adiponectin mRNA expression by three-fold [35]. 

Surprisingly, we have shown that in response to rosiglitazone, there is a decrease in the adiponectin content of mHypo POMC and SH-SY5Y neuronal cells and, this is concomitant with a striking increase in adiponectin secretion in the culture medium as evidenced by Western blot analysis, especially following 48 h of treatment. These findings indicate that the main part of adiponectin expressed by these neurons is secreted in the medium and not stored in the cells. These results are similar to those obtained on adipocytes. Indeed, it has been shown that the secretion of adiponectin in the culture medium by the 3T3-L1-cell line was increased in response to troglitazone, an agonist for PPARγ from the same family as rosiglitazone [32]. These results, therefore, suggest that the expression and secretion of adiponectin by neurons are under the control of PPARγ as in adipocytes.

Besides, in adipocytes, the expression of adiponectin is also increased by FGF21, a hepatokine, via the PPARγ pathway, thus increasing the insulin responsiveness of these cells [21]. Here, we also show that FGF21 treatment increases adiponectin expression in mHypo POMC neurons. This finding may highlight an endogenous regulation of adiponectin at the neuronal level that could orchestrate the neuronal insulin responsiveness. Indeed, we have previously shown that adiponectin and FGF21 signaling pathways are completely abolished by resistin central treatment leading to hypothalamus inflammation and to overall insulin resistance [10]. Furthermore, understanding the mechanisms underlying adiponectin expression and secretion by the MBH could have an important impact in targeting brain neuroinflammation to overcome many neuronal diseases including degenerative diseases and hypothalamic neuroinflammation/insulin resistance that promote metabolic disorders. Indeed, there is an important body of evidence linking for instance the brain impairment of adiponectin signaling within the brain to Alzheimer’s disease [35]. Indeed, here we show that adiponectin partially inhibits the resistin-induced IL6 expression in SIMA9, evidencing its anti-inflammatory action. Furthermore, food restriction led to increased adiponectin plasma levels associated with increased cognition orchestrated by activated AMPK in the hippocampus [36]. The activation of the adiponectin/pAMPK signaling pathway has been described as an anti-inflammatory pathway in BV2 microglia [37]. Thus, we may hypothesize that at the hypothalamic level the expression and secretion of local neuronal adiponectin could be a negative regulator of microglia-induced neuroinflammation.

In summary, we have shown that: 1/adiponectin is expressed by the hypothalamus and neuronal cells; 2/the neuronal expression of adiponectin is altered in a situation of insulin resistance; 3/the expression of adiponectin is induced by FGF21 and by a PPARγ agonist; and 4/adiponectin reduces resistin-induced inflammation.

## Supplementary Materials

The following supporting information can be downloaded at: https://www.mdpi.com/article/10.3390/cells11172725/s1, Figure S1: Table of primers; Figure S2: Adiponectin signal specificity by IHC; Figure S3: Expression of adiponectin in response to 24 or 48 h incubation of Rosiglitazone.
