# Supplementary material for: Evidence for the Neuronal Expression and Secretion of Adiponectin
Source: Cells. 2022 Sep 1;11(17):2725. doi: 10.3390/cells11172725 (PMC9454681; doi:10.3390/cells11172725)
Supplement: Supplementary file 1 [file cells-11-02725-s001.zip › cells-1835134-supplementary.pdf]

cells MDPI

Supplementary Material

Gene	accession number	Forward	Reverse	Amplicon size
mouse adiponectin	NM_009605.4	GGAACTTGTGCAGGTTGGATG	CCCTTCAGCTCCTGTCATTC	171 bp
mouse GAPDH	NM_001289726.1	ATGATGACCCGTTTGGCTCC	GCCATCAACGACCCCTTCAT	269 bp
mouse IL6	NM_031168.2	GCCTTCTTGGGACTGATGCTGGT	TCCTCTGTGAAGTCTCCTCTCCG	77 bp
mouse 18S	NR_003278.3	GTGGGCCTGCGGCTTAAT	GCCAGAGTCTCGTTCGTTATC	171 bp

Figure S1. Table listing all primers used for classic PCR or quantitative PCR with gene accession number, forward and reverse sequences and amplicon size of the transcript.

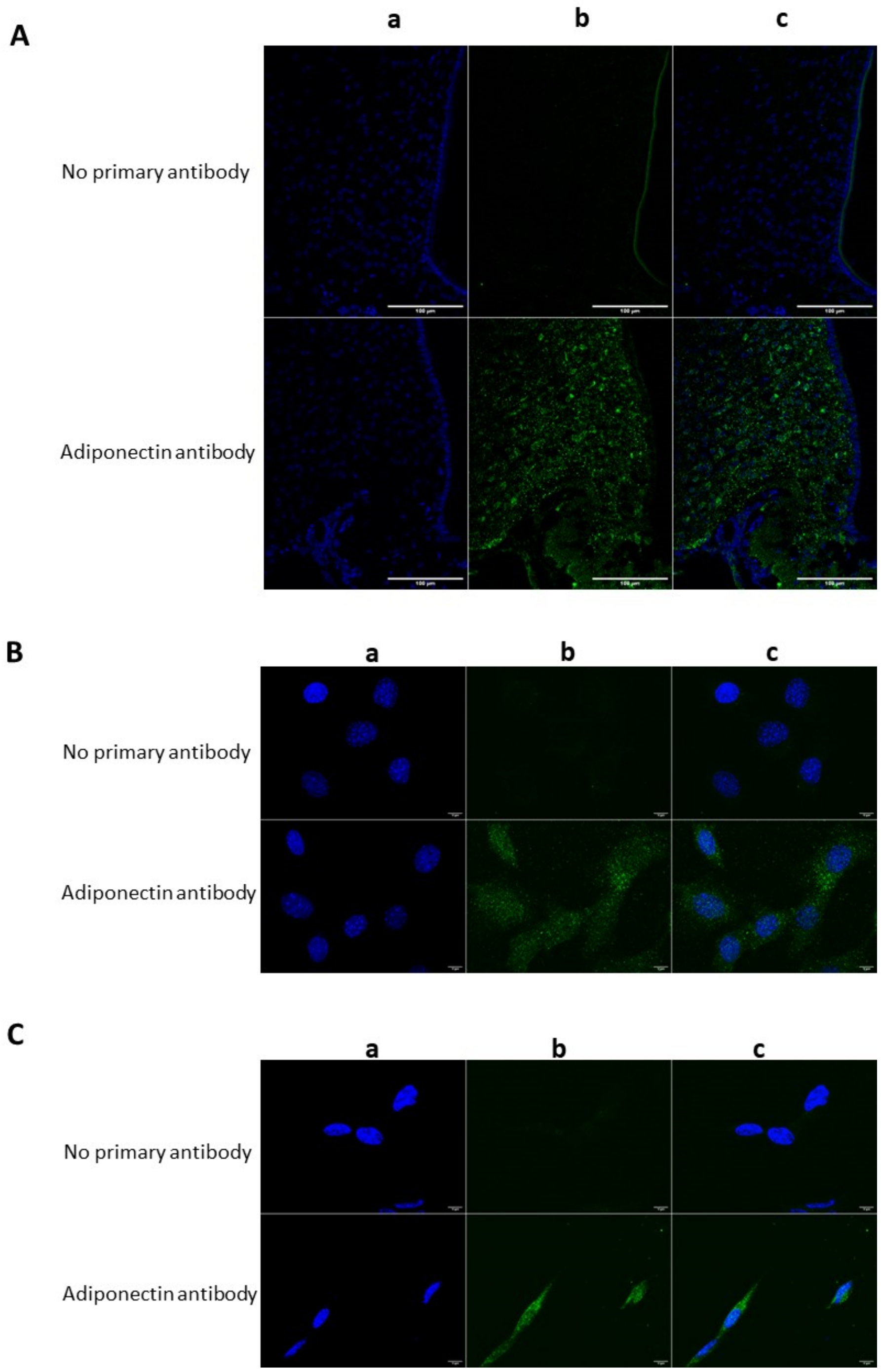

Figure S2. Adiponectin signal is specific in immunohistochemistry and immunocytochemistry assays. (**A**) Adiponectin expression in the hypothalamus medio-basal hypothalamus, more precisely in arcuate nucleus of C57Bl6 mice, (**B**) mouse hypothalamic mHYPO-POMC cells and (**C**) SHSY5Y neuronal cells; a) Immunostaining of a) DAPI (blue), b) Adiponectin (green), and c) merged. Upper panels show absence of green staining when primary antibody is omitted, and presence of specific adiponectin staining when primary antibody is added. All images have been obtained with 25× objective of Epifluorescence-Apotome microscope. Scale bars =100 μm for hypothalamus, 10 μm for cells.

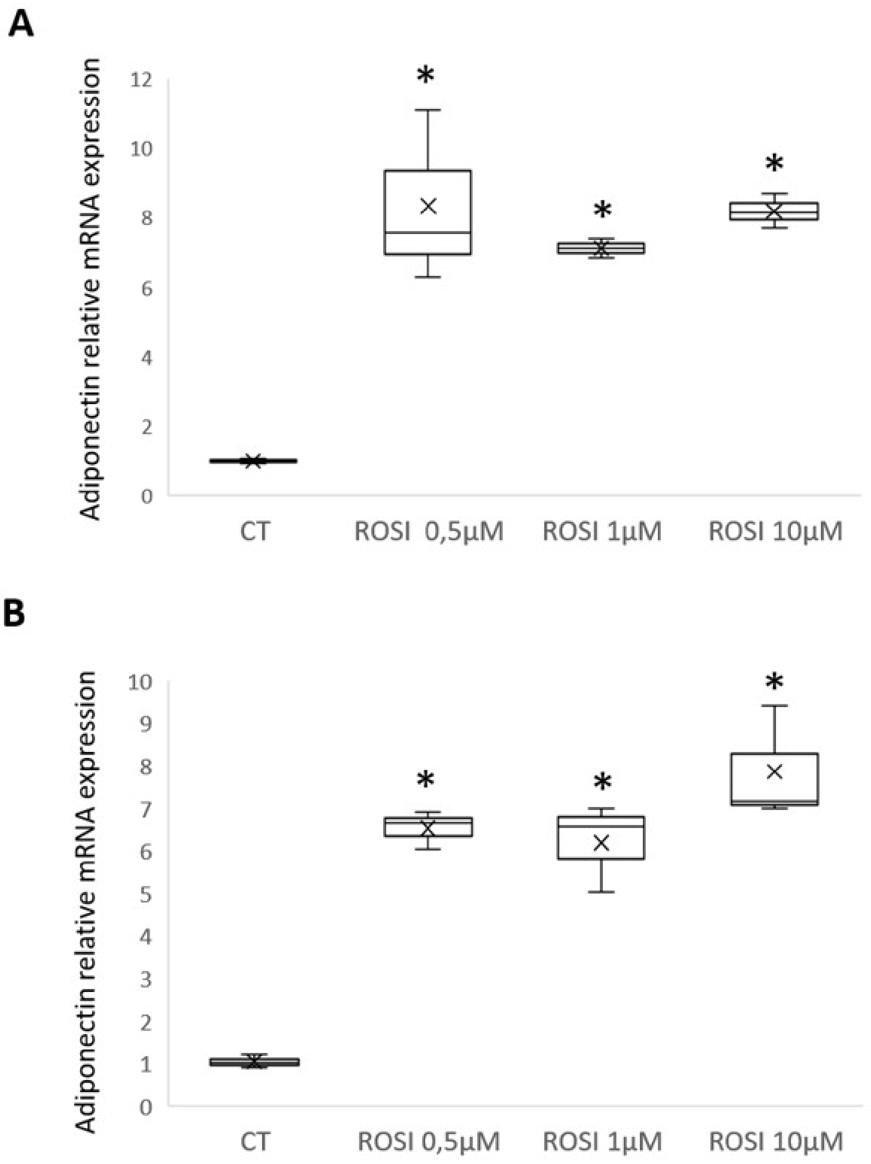

Figure S3. Adiponectin is regulated by rosiglitazone at different doses after 24 h or 48 h stimulation. Adiponectin mRNA expression normalized to GAPDH obtained by RT-qPCR on mHypo-POMC cells treated with different concentrations of Rosiglitazone (0.5 μM, 1 μM and 10 μM) during 24 h (**A**) or 48 h (**B**) (*n* = 1 experiment with triplicates).
